# Sero-epidemiological study of the rotavirus VP8* protein from different P genotypes in Valencia, Spain

**DOI:** 10.1038/s41598-020-64767-x

**Published:** 2020-05-08

**Authors:** Susana Vila-Vicent, Roberto Gozalbo-Rovira, Antonio Rubio-Del-Campo, Cristina Santiso-Bellón, Noemí Navarro-Lleó, Carlos Muñoz, Javier Buesa, Jesús Rodríguez-Díaz

**Affiliations:** Departament of Microbiology, Faculty of Medicine, University of Valencia, Av. Blasco Ibañez 17, 46010 Valencia, Spain

**Keywords:** Virology, Rotavirus

## Abstract

The aims of the present work were to determine the prevalence and titer of serum antibodies against several rotavirus VP8* proteins from different P genotypes in children and adults in Valencia, Spain; and to determine the role of the secretor status (FUT2_G428A_ polymorphism) in the antibody response. The VP8* protein from the P[4], P[6], P[8], P[9], P[11], P[14] and P[25] genotypes were produced in *E. coli*. These proteins were tested with 88 serum samples from children (n = 41, 3.5 years old in average) and from adults (n = 47, 58 years old in average) by ELISA. A subset of 55 samples were genotyped for the FUT2_G428A_ polymorphism and the antibody titers compared. The same subset of samples was also analysed by ELISA using whole rotavirus Wa particles (G1P[8]) as antigen. Ninety-three per cent of the samples were positive for at least one of the VP8* antigens. Differences in the IgG seroprevalence were found between children and adults for the P[4], P[8] and P[11] genotypes. Similarly, significant differences were found between adults and children in their antibody titers against the P[4], P[8], and P[11] VP8* genotypes, having the children higher antibody titers than adults. Interestingly, positive samples against rare genotypes such as P[11] (only in children), P[14] and P[25] were found. While no statistical differences in the antibody titers between secretors and non-secretors were found for any of the tested P genotypes studied, a higher statistic significant prevalence for the P[25] genotype was found in secretors compared to non-secretors. Significant differences in the antibody titers between secretors and non-secretors were found when the whole viral particles from the Wa rotavirus strain (G1P[8]) were used as the antigen.

## Introduction

Rotaviruses are the leading etiologic agent of viral gastroenteritis in infants and young children worldwide^1^. Rotavirus contains segmented, double stranded RNA genome. The rotavirus protein VP7 makes up the outer capsid protein shell and VP4 forms the spikes that emanate through the outer capsid. VP7 is a glycoprotein that determines the G-type antigen while VP4 is a protease-sensitive protein that determines the P-type antigen^2^. The common rotavirus classification involves their genome composition and their serological reactivity, so the scientific community uses the VP7, VP4 and VP6 proteins to classify them. Rotaviruses are classified into 7 groups (A to G) based in the immunological reactivity of the VP6 (middle layer viral protein). Rotaviruses from the groups A to C have been isolated from humans but the most prevalent is the group A.

The two outer capsid protein, VP7 and VP4, are used to classify rotavirus in G and P types (genotypes and/or serotypes). Nowadays we can count more than 36 different G-genotypes and 51 P-genotypes (https://rega.kuleuven.be/cev/viralmetagenomics/virus-classification/rcwg). The most prevalent genotypes in humans infections are G1P[8], G2P[4], G3P[8] and G4P[8]. More recently a classification using the eleven segments of the genome has been settled up. This new classification allows a more in deep knowledge of rotavirus epidemiology to better study the occurrence of genetic reassortants^3^.

**Figure 1:**
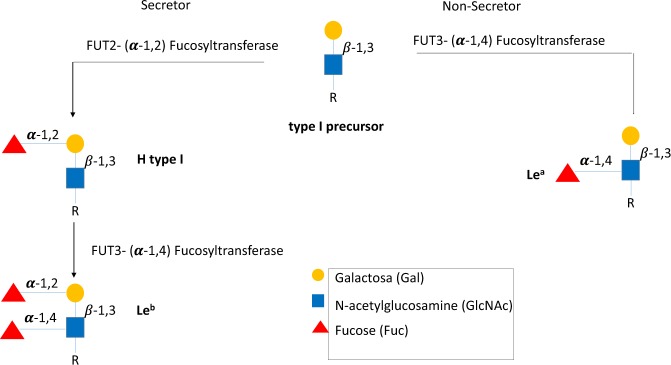
HBGA biosynthesis pathway of the type I precursor. The secretor status depends on the presence of FUT2 enzyme that adds a fucose in α-1,2 position of the galactose from the disaccharide precursor. Non-secretor individuals show a lack of functionality of this enzyme and they are not able to synthesise the H type antigen. In this case, FUT3/4 adds a fucose in the α-1,4 position leading to the generation of Le^a^ antigen.

The spike protein VP4 is processed by proteolytic cleavage into two subunits, VP5* and VP8*. VP8* is mainly involved in the virus - cell attachment process, while VP5* is involved in the translocation of the virus into the cytoplasm of host cells. For more than 30 years sialic acid was considered the glycoside receptor for animal rotaviruses^4^. Interestingly, human strains were considered sialic acid independent. The discovery that the VP8* from human rotavirus strains also interacts with sugars, components of the histo-blood group antigens (HBGAs), broke the paradigm of the understanding of rotavirus biology, showing that the first steps of infection of human rotaviruses are mediated by specific carbohydrate-virus interactions^5,6^. HBGAs synthesis occurs by a sequential addition of monosaccharides to a disaccharide precursor. The precursor contains the sugars galactose and N-acetylglucosamine (GlcNAc) linked by a β-1,3 or β-1,4 linkage. Depending on the linkage the sugars are classified as type I containing β-1,3 linkage whereas type II includes β-1,4 linkage. To synthesize the H antigen is needed the addition of a fucose in the α-1,2 position by the enzyme FUT2. Lewis antigens are synthesised by the addition of a fucose residue in the position α-1,4 or α-1,3 to the terminal GlcNAc or H type I and II precursors respectively to create the different Lewis a, b, x and y antigens^7^. The secretor status is defined by the FUT2 gene, non-secretor individuals are those that lack functionality in both FUT-2 alleles and subsequently do not express H-antigen structures (Fig. 1).

Several studies suggested that non-secretor status reduces the susceptibility to RV infections mostly related to the P[8], P[4] and P[6] rotavirus genotypes^8–10^. This is true in our area where the non-secretor infants are less prone to suffer symptomatic rotavirus infections^11^.

Sero-epidemiological studies are of importance to elucidate the different viral agents and genotypes that are circulating in a particular area, for this reason the main aim of this work was to determine the serum antibody prevalence and titer to a panel of VP8* proteins from different rotavirus genotypes (P[4], P[6], P[8], P[9], P[11], P[14] and P[25]) in children and adults. The second aim was to determine if the secretor status (FUT2_G428A_ polymorphism) was related to differential antibody response as a measure of susceptibility to the different rotavirus genotypes utilised here.

## Results

### Expression and purification of the rotavirus VP8* protein from several genotypes

**Figure 2:**
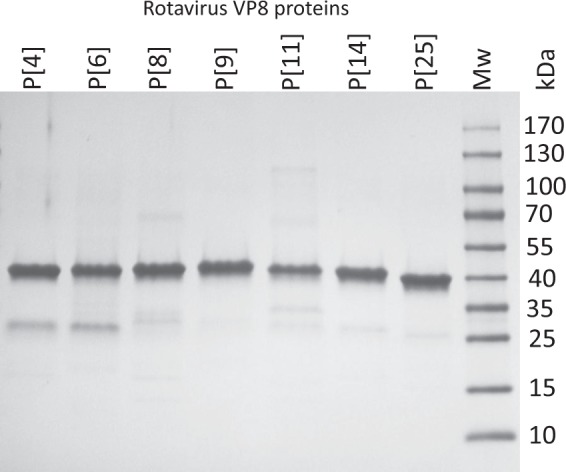
Coomassie blue stained 12% SDS-PAGE gel showing the different GST::VP8* proteins used in this work. The genotype of each one of the proteins is indicated in the upper part of the gel. The molecular weights (kDa) of the marker (Mw) are indicated on the right of the gel.

The VP8* protein from P[4], P[6], P[8], P[9], P[11], P[14] and P[25] genotypes were produced in *E. coli* as described in the Material and methods section. The purified proteins were analysed by SDS-PAGE in a 12% polyacrylamide gel (Fig. 2) and confirmed by Western blot using an anti-GST antibody. The purity of each protein reached 90% and their sizes were as expected.

### Prevalence of serum anti-rotavirus VP8* antibodies

The results of the present study show that ninety-three per cent of the serum samples were positive for at least one of the VP8* antigens (82/88). Different prevalence was found for each of the genotypes both in adults and children (Table 1). The higher prevalence was found against the P[4] and P[8] genotypes, while the lower prevalence was against the P[11] genotype. Considering the total prevalence, no differences were found among children 88% (36/41) and adults 98% (46/47) (Table 1). Most interesting, the prevalence to P[4], P[8] and P[11] genotypes was higher in children than in adults (*P* < *0.05*) (Table 1). Contrarily, not significant differences were found for any of the other tested genotypes.

**Table 1:** Seroprevalence of anti-rotavirus VP8* proteins in adults and children in Valencia, Spain.

VP8* Genotype	Adults	Children	χ^2^ test
P[4]	26/47 (56%)	33/41 (80%)	*P* = 0.01*
P[6]	32/47 (68%)	28/41 (68%)	*P* = *0.98*
P[8]	23/47 (49%)	29/41 (70%)	*P* = 0.04*
P[9]	16/47 (34%)	22/41 (53%)	*P* = *0.06*
P[11]	0/47 (0%)	11/41 (27%)	*P* = 0.0006*
P[14]	27/47 (57%)	18/41 (44%)	*P* = *0.2*
P[25]	14/47 (30%)	20/41 (49%)	*P* = *0.06*
Any genotype	46/47 (98%)	36/41 (88%)	*P* = *0.6*

The significance levels are indicated for each genogroup*. P* < *0.05* is considered statistically significant (*).

**Figure 3:**
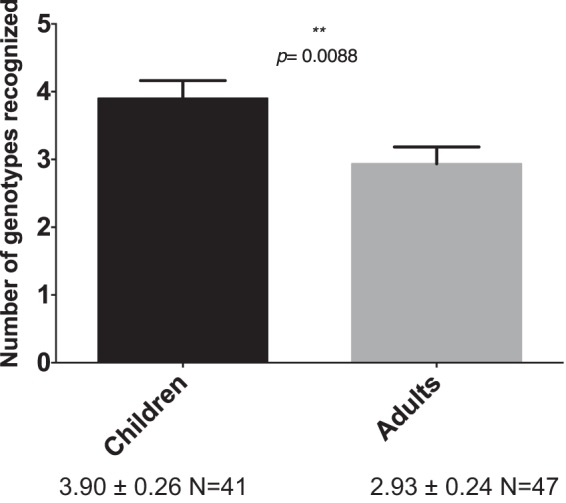
Number of genotypes recognised by serum samples from children and adults. The children population recognised a mean 3.90 genotypes while the adult population recognize a lower number of genotypes (2.93). *P*-value indicates that the difference is significant between both populations (*P* < *0.05)*.

The number of different genotypes recognised by a single serum sample was also studied. The results show that children serum samples recognize a higher number of genotypes (3.90 ± 0.26) compared to adults (2.94 ± 0.24) (*P* < 0.05) (Fig. 3).

### Serum antibody titers to rotavirus VP8* proteins

**Figure 4:**
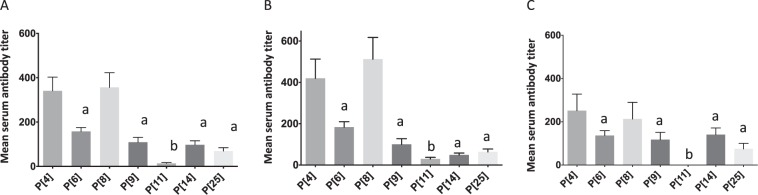
Mean values of serum antibody titers from adults and children (n = 88) in panel A, from only children (n=41) in panel B and from adults (n=47) in panel C, against each rotavirus VP8* genotype. The error bars indicate the standard error of the mean (SEM). Statistical analyses (one way ANOVA) were performed and show that in the three populations the antibodies against P[6], P[9], P[14] and P[25] are significantly lower than against P[4] and P[8] (*P* < *0.05*^*a*^). The lower antibody titers were found to the P[11] genotype compared to any of the other genotypes (*P* < *0.0001*^*b*^).

The serum samples from the present study had different antibody titers against each of the different VP8* genotype studied (Fig. 4A). The higher antibody titers were detected against the P[4] and P[8] genotypes and the lower antibody titers towards the P[11] genotype (*P* < 0.0001). These differences were observed both among children and adults (Fig. 4B,C respectively).

**Figure 5:**
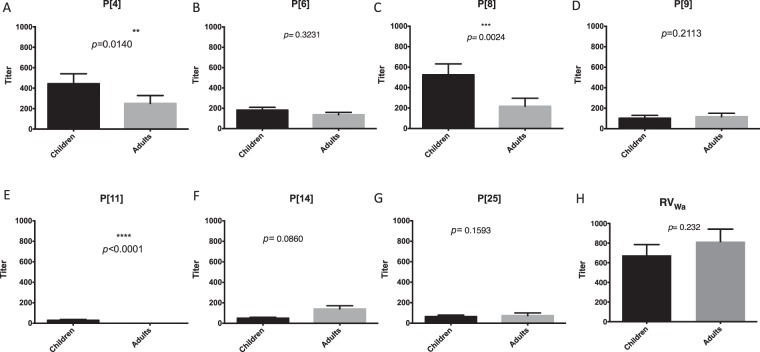
Comparison of the rotavirus VP8* antibody titers in children and adults. From A to G panels, the IgG titer against each VP8* protein is shown. Y axis represents the mean titer of IgG reached in the serum samples from adults or children against the VP8* protein. The error bars represent the standard error of the mean (SEM). Only for P[4], P[8] and P[11] the differences obtained between adults and children were statistically significant (*P* < 0.05). The H panel shows the results using the whole rotavirus particle from the Wa strain, a P[8] strain.

The comparison of the antibody titers between adults and children (Fig. 5), showed that the children have higher titers against P[4], P[8] and P[11] genotypes compared to adults (*P* = *0.014, P* = *0.0024 and P* < *0.0001 respectively*). The antibody titers to P[6], P[9] and P[25] genotypes were similar in both groups, and the titer against P[14] genotype was higher in the adults group, but not significative. Also, no differences were found between children and adults when the complete rotavirus Wa viral particle was used as antigen (Fig. 5H).

### Secretor status and antibody response to the rotavirus VP8* proteins

**Figure 6:**
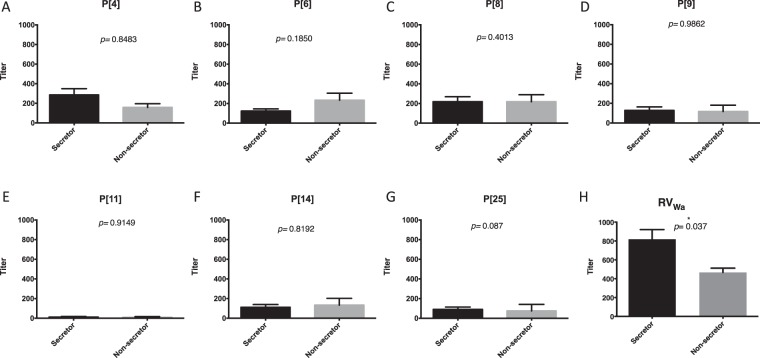
Relationship between the secretor status and the antibody titer against each VP8* genotype, panels A to G. The data analysed include 43 secretor and 12 non-secretor individuals. Statistical analyses were performed (U Mann Whitney to unpaired samples) to determine the differences between the antibodies titers. The H panel shows the results using the whole rotavirus particle from the Wa strain, a P[8] strain. The error bars represent the standard error of the mean (SEM). The samples did not show any significant differences between these two populations for the VP8* proteins (*P* > 0.05). Significant differences (*P* = 0.037) were found when the complete rotavirus particle was used (panel H).

**Figure 7:**
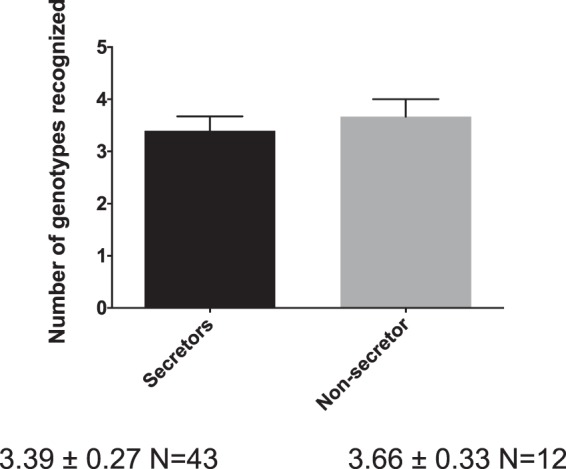
Number of genotypes recognised by serum antibodies. In secretor individuals (n = 43) the data show that these serum samples contain antibodies against 3.3 genotypes as a mean. Besides, non-secretor population serum samples (n = 12) were able to recognize 3.6 genotypes. These data were analysed statistically (non-parametric test from unpaired samples) to confirm that there are non-differences between secretor and non-secretor (*P* > *0.05)*. Error bars represent the standard mean error (SEM).

Genomic DNA was extracted directly from serum samples and the FUT2 genotype could be determined in 55 of them. The remaining 33 samples did not present enough DNA or it was too damaged to perform the genotyping. The Supplementary Table S1 shows a compilation of the antibody response and secretor status of each of the analysed samples. Of the 55 samples 43 were secretor positive (78%) and 12 were secretor negative (21%); these percentages are similar to those previously published by us for the general population in the study area^11,12^. Many comparations were stablished to study the differences in prevalence (Table 2), in antibody titers (Fig. 6) and in the number of different genotypes recognised between secretors and non-secretors (Fig. 7). Interestingly, P[25] genotype showed a higher prevalence in secretors compared to non-secretors (*P* = *0.045)*. The other genotypes showed similar frequencies between these two populations (Table 2).

**Table 2:** Seroprevalence of anti-rotavirus VP8* proteins in secretors and non-secretors in Valencia, Spain.

VP8* Genotype	Secretors	Non-secretors	χ^2^ test
P[4]	28/43 (65%)	10/12 (83%)	*P* = *0.22*
P[6]	26/43 (60%)	9/12 (75%)	*P* = *0.35*
P[8]	23/43 (53%)	9/12 (75%)	*P* = *0.18*
P[9]	20/43 (46%)	6/12 (50%)	*P* = *0.83*
P[11]	4/43 (9%)	1/12 (8.3%)	*P* = *0.93*
P[14]	24/43 (56%)	6/12 (50%)	*P* = *0.72*
P[25]	21/43 (49%)	2/12 (16%)	*P* = 0.045*
Any genotype	39/43 (90%)	12/12 (100%)	*P* = *0.6*

The significance levels are indicated for each genogroup*. P* < *0.05* is considered significative (*).

The comparison of the antibodies titers between secretors and non-secretors did not showed any difference between both groups (Fig. 6). Moreover, the genotypes recognised by each serum sample in secretor (3.39 ± 0.28) and non-secretor (3.66 ± 0.33) population did not show significant differences (*P* = *0.626)* (Fig. 7). Interestingly, when the whole viral particle of the rotavirus Wa strain, a P[8] strain, was used as antigen in the ELISA experiments, significant differences were found between secretors and non-secretors (*P* = *0.037*) (Fig. 6H).

## Discussion

Rotaviruses are a major cause of acute gastroenteritis in children under 5 years of age worldwide and rotavirus infections are responsible of approximately 128.000 deaths per year^13^. The prevalence is similar in developed and developing countries, but the majority of deaths occur in developing countries, where rotavirus vaccines are not available or are less efficient than in developed countries^14^. In the present study the reactivity of 88 serum samples from adults and children was analysed against seven different rotavirus VP8* proteins from different P genotypes, some from common rotavirus genotypes, P[4], P[6], P[8] and P[9] but others from uncommon such as P[11], P[14] and P[25]^15^. To our knowledge, this is the first time that seven different GST::VP8* protein have been used in a sero-epidemiological study, providing a high capacity of screening.

Our study reveals the presence of serum IgG antibodies against all the genotypes assessed, indicating that the exposure to rotavirus is habitual in all age stages. The most common G genotypes distributed worldwide are represented by G1, G2, G3, G4, G9 and G12 in combination with mostly P[8] and P[4] (approximately 88%)^16^. These genotypes are responsible for almost 90% of the rotavirus infections globally^15^ and also in our study area^11^. In two recent publications addressing the molecular epidemiology of human rotavirus in Valencia (Spain), it was shown that 98.5% of the samples were from the P[8] genotype and only 1.5% belonged to the P[4] genotype^11,17^. None of the other genotypes assayed on the present study were found in clinical samples (P[6], P[9], P[11], P[14], P[25]). Accordingly, the results of the present study show a higher prevalence and titers to the P[8] and P[4] genotypes. Interestingly, the P[11] genotype was only recognised by the serum samples from children, reinforcing the evidence that P[11] genotype has a higher age restriction than other genotypes, probably due to the ability of this genotype to recognize non fucosylated receptors^18^.

Interestingly, antibodies against all the tested genotypes were found, indicating that even if they are not found in clinical samples these viruses are also circulating in the population at a subclinical level. Some of the less frequent genotypes including P[11], P[14] and P[25] are considered reassortant strains between animal and human rotaviruses or even transmitted directly from animals to humans^19^. This fact might explain a lower viral fitness in the human host causing less severe or asymptomatic infections, in fact the RotaTeq vaccine is a bovine – human reassortant.

Histo-blood group antigens (HBGAs) are found in the gastric mucine and in the surface of the epithelial cells in the gut. Many investigations have shown that VP8* from rotavirus are able to recognize different HBGAs in a genotype depending manner^7^. The P[9] and P[14] genotypes belong to genogroup III and are able to recognize the A blood group antigen^20^. P[19] genotype (a genotype that infects pigs and humans), belongs to the genogroup II and binds to the H antigen and to the mucin core 2 glycan^21^. In the same manner, P[4], P[6] and P[8] that also belong to the genogroup II, are able to recognize H type related antigens and the type I precursor^5,22^. Finally the P[11] genotype interacts with the H type II precursor molecule^18^. Several studies have demonstrated a different susceptibility to rotavirus infections depending in the ability to produce the H antigen. In a recent epidemiological study performed in the same geographic region it was shown that 98% of the clinical samples belonged to secretor positive children indicating that rotaviruses from the P[8] and P[4] genotypes infect more severally these children^11^.

The serology presented here shows that the antibody titers against all the VP8* from the different P types do not depend on the secretor status. Interestingly, when the whole rotavirus Wa particle was used, significant higher titers against rotavirus was found in secretors, compared to non-secretors. These data confirm a previous report of higher anti-rotavirus titers in secretor individuals in Sweden^23^. It is important to mention that structural and non-structural proteins such as VP2,VP6 and NSP2 are immunodominant^24^, while VP8* is poorly recognised by serum antibodies^25^. These differences in immunodominance might explain why the differences between secretors and non-secretors can only be observed when the whole viral particle containing VP2 and VP6 (plus VP4 and VP7) are used as the antigen to determine antibody titers. These results are highly relevant, in the context of vaccine development, since a VP8* subunit vaccine is under development^26^. Common vaccines such as Rotarix (G1P[8]) and RotaTeq (G1-G4 P[8]) are protective against a broad range of rotavirus G and P genotypes, even against those not included in the vaccines^27^. This might be due to the cross immunity raised to the immunodominant VP6 and VP2 proteins that are more conserved in group A rotaviruses, independently of the G and P genotypes^2^. This might explain, as well, the level of protection achieved with the Rotavac vaccine which is composed of a G9P[11] genotype^28^. In regard to the VP8* subunit vaccine, it has been demonstrated that the VP8* protein can elicit neutralising and protective antibodies^29–31^. However it has also been demonstrated that the protection is homotypic^31^, indicating that this vaccine might only protect against the rotavirus carrying the genotypes included in the formulation. On the other hand, one of the advantages of the VP8* subunit vaccine is that it is administered by the parenteral route, shortcutting the differences in the vaccine uptake observed between secretors and non-secretors^26^.

Our research group has recently elucidated the molecular bases of P[8]::H antigen interactions and we demonstrated that rotavirus can bind both the H antigen and its precursor (H-type I, Lacto-N-Biose) but this interaction is weaker when the fucose is lacking^5^ reinforcing the idea that the secretor status is a key host factor in rotavirus infections. Furthermore, when the VP8* binding pocket was compared between different genotypes and lineages it was found that the VP8* from the Rotarix vaccine possesses a mutation (L167F) that disrupts the binding pocket providing an explanation for the attenuation of this strain^5^. More surprisingly, we found significative differences in the prevalence of antibodies against the P[25] genotype in secretors and non-secretors. This genotype is known to bind to the blood group A antigen^5^. The presence of the A group antigen depends on the A enzyme coded in the ABO locus. The A enzyme utilizes the H antigen as substrate, this means that secretor negative individuals are unable to synthesize the A blood group antigen in their mucosae, giving an explanation to the lower susceptibility of non-secretors to a virus that binds to the A antigen instead of the H antigen.

As a conclusion we show here the usefulness of sero-epidemiological studies to understand the circulation of common (P[4] and P[8]) and uncommon (P[6], P[9], P[11], P[14], P[25]) rotavirus genotypes in a given population and showed for the first time a relationship between the secretor status and the susceptibility to the rotaviruses of the P[25] genotype.

## Material and Methods

### Expression and purification of recombinant VP8*

The VP8* protein from P[4], P[6], P[8], P[9], P[11], P[14] and P[25] genotypes were produced as described previously^5^. Briefly, the different *E.coli* clones were cultured in LB medium with ampiciline and kanamycine (final concentration 100 μg/ml and 25 μg/ml respectively) at 37 °C with vigorous shacking until the culture reached an O.D._600_ = 0.5, then the cultures were induced with IPTG to a final concentration of 0.1 mM. The cultures were maintained at 25 °C overnight. After induction, the cells were recovered by centrifugation at 5,000 × g for 20 minutes. The cells from 1 L of culture medium were lysed in 15 mL of lysis buffer (PBS with 0.5% of protease inhibitor cocktail (Sigma), lysozyme 1 mg/ml, 125U DNAseI (Thermo Fischer Scientific) and 0.5 mM DTT). The lysates were centrifuged at 15,000 × g for 30 minutes and the supernatants were filtered through 0.45 μm syringe filters (Millipore). The proteins were purified in an Äkta prime FPLC system (GE, Healthcare) carrying a GST-Trap (GE, Healthcare) column in PBS. The elution buffer consisted in 50 mM Tris-HCl, 10 mM glutathione, pH 8.0. GST::VP8* proteins were desalted using a HiTrap Desalting Column (GE, Healthcare) in the same Äkta prime FPLC system according to manufacturer’s protocol.

### Sample collection

Serum samples were obtained from Hospital Clínico Universitario de Valencia. A total of 88 serum samples were collected from adults and children from general population. Samples were from 3.5 years old kids in average and from 58 years old adults in average. The information about the samples is shown in Supplementary Table 1.

### Ethic statement

This study was conducted with the approval of the Ethics Committee of the University of Valencia (code H1544010468380). The ethic committee waived the need of informed consent since human blood samples from Hospital Clínico Universitario de Valencia were anonymised previously to their inclusion in the present study. The study was performed following the declaration of Helsinki on ethical principles for medical research involving human subjects.

### Determination of anti-VP8* IgG antibodies in serum samples

IgG antibodies from 88 serum samples were analysed by ELISA against seven different VP8* genotypes, P[4], P[6], P[8], P[9], P[11], P[14] and P[25] as previously described with modifications^32^. ELISA plates were coated by each GST::VP8* proteins (P[4], P[6], P[8], P[9], P[11], P[14] and P[25]). The purified proteins were diluted in carbonate/bicarbonate buffer pH 9.6 at 1μg/ml. One-hundred μl of solution was added to each well and incubated overnight at 4 °C. In parallel, wells in the same plates were coated with the GST protein (1 μg/ml) as reference protein to establish the cut-off for each sample. The plates were blocked by adding 200 μl PBS containing 0.05% Tween 20 (PBS-T) and 3% BSA for 1 hour at 37 °C. After blocking, the plates were washed three times with PBS-T. The serum samples were serially diluted two-fold from 1/100 to 1/6,400 and were added to the plates in triplicate and incubated for 1 hour at 37 °C. After three washes a goat anti-human-IgG antibody (1:1,000) conjugated with HRP was added for 1 hour at 37 °C. One hundred μl of the o-phenylenediamine (OPD-Fast reagent. Sigma) were added to each well and the reaction was stopped adding 50 μL of 3 M of H_2_SO_4_ and incubating for 10 minutes. The absorbance was recorded at 492 nm. The dilution was considered positive when the absorbance in the GST::VP8* well was higher than the same dilution against GST plus three standard deviations.

### Determination of anti-Wa rotavirus IgG antibodies in serum samples

IgG antibodies from the 55 serum samples that could be FUT2-genotyped were analysed by ELISA against the whole rotavirus from the Wa strain, a human G1P[8] genotype. Rotavirus particles were purified as previously described^5^ and ELISA plates were coated with 100 μl per well of a solution containing 2.5 μg/ml of purified virus in carbonate/bicarbonate buffer pH 9.6. The ELISA was performed as described above but, in this case, the cut-off was calculated in wells coated with 100 μl of BSA at 1 μg/ml in carbonate/bicarbonate buffer pH 9.6. The dilution was considered positive when the absorbance in the rotavirus Wa well was higher than the same dilution against BSA plus three standard deviations.

### Genotyping secretor status

Genomic DNA was extracted from serum samples with a commercial kit (JetFlex Genomic DNA Purification kit, Genomed, Vilnius, Lithuania) following the manufacturer’s instructions. The FUT2_G428A_ polymorphism was determined by PCR and RFLP as previously described^33,34^.

### Statistical analyses

Categorical data were analysed using the χ2 test. Differences in the antibody response to all the VP8* tested genotypes was analysed with the one-way anova test. Differences in the mean titers and in mean number of genotypes between two groups were analysed by the non-parametrical test Mann-Whitney U. The analyses were performed with the GraphPad Prism 6 software for MacOsX. *P* values lower than 0.05 were considered statistically significant.

## Supplementary information

Supplementary information.
