# Supplementary material for: Sero-epidemiological study of the rotavirus VP8* protein from different P genotypes in Valencia, Spain
Source: Sci Rep. 2020 May 8;10:7753. doi: 10.1038/s41598-020-64767-x (PMC7210269; doi:10.1038/s41598-020-64767-x)
Supplement: Supplementary file 1 — Supplementary information. [file 41598_2020_64767_MOESM1_ESM.docx]

**Sero-epidemiological study of the rotavirus VP8* protein from different P genotypes in Valencia, Spain**

Susana Vila-Vicent^Ψ^, Roberto Gozalbo-Rovira^Ψ^, Antonio Rubio-Del-Campo, Cristina Santiso-Bellón, Noemí Navarro-Lleó, Carlos Muñoz, Javier Buesa and Jesús Rodríguez-Díaz*

Departament of Microbiology, Faculty of Medicine, University of Valencia, Av. Blasco Ibañez 17, 46010 Valencia, Spain

^Ψ^These two authors contributed equally

*To whom correspondence should be addressed:

Jesús Rodríguez-Díaz

Department of Microbiology, School of Medicine, University of Valencia, Avda. Blasco Ibáñez 17, 46010 Valencia, Spain

Email: Jesus.rodriguez@uv.es

Phone: +34 963864903; Fax: +34 963864960

Supplementary table 1

| **Sample** | **Age** | **P[4]** | **P[6]** | **P[8]** | **P[9]** | **P[11]** | **P[14]** | **P[25]** | **RV_Wa_** | **Secretor status** |
| --- | --- | --- | --- | --- | --- | --- | --- | --- | --- | --- |
| 1 | 3 | 0 | 0 | 0 | 100 | 0 | 0 | 0 | UD | UD |
| 2 | 3 | 400 | 0 | 400 | 0 | 0 | 0 | 0 | 400 | Se+se- |
| 3 | 3 | 100 | 100 | 400 | 200 | 0 | 200 | 400 | 800 | Se+se- |
| 4 | 3 | 0 | 0 | 0 | 0 | 0 | 0 | 0 | UD | UD |
| 5 | 3 | 400 | 0 | 0 | 100 | 0 | 100 | 100 | 800 | se-se- |
| 6 | 3 | 200 | 100 | 800 | 0 | 0 | 100 | 100 | 3200 | Se+se- |
| 7 | 3 | 200 | 0 | 800 | 100 | 100 | 0 | 0 | 400 | se-se- |
| 8 | 3 | 200 | 0 | 1600 | 100 | 0 | 100 | 0 | 1600 | Se+Se+ |
| 9 | 3 | 100 | 0 | 3200 | 100 | 0 | 100 | 0 | 400 | se-se- |
| 10 | 4 | 0 | 0 | 0 | 100 | 0 | 0 | 100 | 400 | Se+Se+ |
| 11 | 4 | 0 | 0 | 200 | 100 | 100 | 0 | 100 | 800 | Se+Se+ |
| 12 | 4 | 400 | 0 | 400 | 100 | 0 | 0 | 0 | 800 | Se+se- |
| 13 | 4 | 100 | 0 | 0 | 800 | 0 | 200 | 400 | 800 | Se+se- |
| 14 | 4 | 400 | 100 | 800 | 100 | 0 | 0 | 100 | 800 | Se+se- |
| 15 | 5 | 0 | 0 | 0 | 0 | 0 | 0 | 0 | 800 | Se+se- |
| 16 | 5 | 100 | 100 | 0 | 0 | 0 | 0 | 0 | UD | UD |
| 17 | 2 | 400 | 200 | 400 | 0 | 0 | 100 | 100 | 400 | Se+se |
| 18 | 2 | 400 | 0 | 0 | 400 | 0 | 0 | 100 | 400 | Se+se |
| 19 | 3 | 800 | 200 | 1600 | 400 | 0 | 400 | 200 | UD | UD |
| 20 | 3 | 800 | 100 | 400 | 0 | 0 | 400 | 0 | 400 | Se+Se+ |
| 21 | 4 | 400 | 200 | 0 | 800 | 0 | 400 | 400 | 400 | Se+Se+ |
| 22 | 4 | 1600 | 100 | 800 | 0 | 0 | 800 | 100 | 400 | Se+Se+ |
| 23 | 4 | 0 | 100 | 0 | 0 | 0 | 800 | 0 | 400 | se-se- |
| 24 | 5 | 400 | 200 | 100 | 0 | 0 | 0 | 0 | 400 | Se+se- |
| 25 | 5 | 0 | 0 | 0 | 0 | 0 | 400 | 0 | UD | UD |
| 26 | 5 | 200 | 400 | 100 | 0 | 0 | 0 | 0 | 400 | se-se- |
| 27 | 3 | 200 | 0 | 0 | 0 | 0 | 0 | 0 | UD | UD |
| 28 | 3 | 0 | 400 | 100 | 0 | 0 | 0 | 0 | 400 | se-se- |
| 29 | 4 | 0 | 200 | 0 | 0 | 0 | 0 | 0 | UD | UD |
| 30 | 4 | 0 | 100 | 0 | 100 | 0 | 100 | 0 | UD | UD |
| 31 | 5 | 0 | 0 | 0 | 0 | 0 | 0 | 0 | UD | UD |
| 32 | 4 | 100 | 800 | 400 | 800 | 0 | 400 | 800 | 400 | se-se- |
| 33 | 5 | 200 | 100 | 0 | 0 | 0 | 100 | 100 | 400 | Se+se- |
| 34 | 5 | 100 | 0 | 100 | 0 | 0 | 0 | 0 | 400 | Se+se- |
| 35 | 2 | 0 | 100 | 200 | 0 | 0 | 0 | 0 | 400 | Se+se- |
| 36 | 4 | 0 | 0 | 0 | 0 | 0 | 0 | 0 | 400 | Se+Se+ |
| 37 | 1 | 200 | 100 | 100 | 0 | 0 | 0 | 0 | UD | UD |
| 38 | 4 | 0 | 100 | 0 | 0 | 0 | 0 | 0 | 400 | Se+Se+ |
| 39 | 3 | 400 | 0 | 0 | 0 | 0 | 100 | 0 | UD | UD |
| 40 | 2 | 0 | 0 | 0 | 0 | 0 | 0 | 0 | 1600 | Se+Se+ |
| 41 | 3 | 0 | 100 | 0 | 0 | 0 | 0 | 0 | UD | UD |
| 42 | 25 | 0 | 400 | 0 | 0 | 0 | 0 | 0 | 1600 | Se+se- |
| 43 | 28 | 0 | 100 | 100 | 0 | 0 | 100 | 0 | 1600 | Se+se- |
| 44 | *33* | 100 | 400 | 400 | 800 | 0 | 200 | 200 | 800 | Se+se- |
| 45 | *35* | 400 | 200 | 100 | 0 | 0 | 100 | 0 | 800 | Se+Se+ |
| 46 | *40* | 0 | 0 | 0 | 0 | 0 | 0 | 0 | 800 | Se+Se+ |
| 47 | *44* | 100 | 0 | 0 | 100 | 0 | 100 | 0 | 400 | Se+Se+ |
| 48 | *44* | 100 | 100 | 0 | 200 | 0 | 100 | 0 | 800 | Se+Se+ |
| 49 | *51* | 0 | 0 | 0 | 0 | 0 | 100 | 100 | 1600 | Se+Se+ |
| 50 | *52* | 0 | 100 | 0 | 0 | 0 | 200 | 100 | 800 | Se+Se+ |
| 51 | *52* | 100 | 0 | 0 | 0 | 0 | 100 | 0 | 400 | Se+se- |
| 52 | 54 | 0 | 200 | 0 | 200 | 0 | 200 | 100 | UD | UD |
| 53 | *54* | 100 | 100 | 200 | 0 | 0 | 100 | 0 | 400 | se-se- |
| 54 | *54* | 400 | 100 | 100 | 0 | 0 | 0 | 0 | 400 | se-se- |
| 55 | *54* | 400 | 0 | 800 | 0 | 0 | 100 | 0 | 400 | Se+se- |
| 56 | *55* | 400 | 200 | 200 | 100 | 0 | 800 | 800 | 400 | Se+se- |
| 57 | 55 | 0 | 0 | 100 | 100 | 0 | 100 | 200 | UD | UD |
| 58 | 56 | 0 | 100 | 0 | 0 | 0 | 0 | 0 | UD | UD |
| 59 | *61* | 0 | 200 | 100 | 800 | 0 | 100 | 0 | 800 | Se+se- |
| 60 | *61* | 0 | 400 | 0 | 200 | 0 | 100 | 200 | 400 | Se+Se+ |
| 61 | *61* | 100 | 100 | 200 | 200 | 0 | 100 | 0 | 400 | se-se- |
| 62 | *62* | 200 | 0 | 200 | 100 | 0 | 0 | 0 | 400 | Se+se- |
| 63 | 72 | 3200 | 200 | 3200 | 200 | 0 | 0 | 0 | UD | UD |
| 64 | *63* | 200 | 400 | 0 | 0 | 0 | 0 | 0 | 400 | Se+Se+ |
| 65 | *63* | 200 | 400 | 0 | 100 | 0 | 0 | 100 | 400 | Se+Se+ |
| 66 | 63 | 100 | 400 | 100 | 100 | 0 | 0 | 0 | UD | UD |
| 67 | *64* | 200 | 400 | 400 | 0 | 0 | 0 | 0 | 400 | se-se- |
| 68 | *64* | 100 | 400 | 100 | 100 | 0 | 0 | 0 | 800 | se-se- |
| 69 | 64 | 400 | 200 | 200 | 100 | 0 | 100 | 100 | UD | UD |
| 70 | 65 | 200 | 200 | 100 | 0 | 0 | 100 | 0 | UD | UD |
| 71 | *65* | 0 | 400 | 0 | 100 | 100 | 100 | 100 | 1600 | Se+Se+ |
| 72 | *66* | 800 | 400 | 400 | 200 | 100 | 100 | 100 | 800 | Se+Se+ |
| 73 | *67* | 1600 | 100 | 400 | 0 | 0 | 0 | 100 | 3200 | Se+se- |
| 74 | 70 | 800 | 100 | 400 | 0 | 0 | 0 | 0 | UD | UD |
| 75 | 70 | 3200 | 400 | 1600 | 200 | 100 | 0 | 100 | UD | UD |
| 76 | *70* | 1600 | 100 | 100 | 100 | 200 | 100 | 100 | 400 | Se+Se+ |
| 77 | 71 | 1600 | 200 | 800 | 0 | 0 | 0 | 100 | UD | UD |
| 78 | 71 | 800 | 100 | 400 | 0 | 0 | 0 | 0 | UD | UD |
| 79 | 72 | 800 | 400 | 1600 | 0 | 0 | 0 | 100 | UD | UD |
| 80 | 73 | 800 | 400 | 800 | 800 | 0 | 0 | 100 | UD | UD |
| 81 | 74 | 400 | 400 | 1600 | 200 | 0 | 100 | 0 | UD | UD |
| 82 | 75 | 800 | 400 | 1600 | 200 | 0 | 100 | 0 | UD | UD |
| 83 | 76 | 100 | 100 | 0 | 0 | 100 | 100 | 100 | UD | UD |
| 84 | 76 | 100 | 400 | 100 | 0 | 100 | 100 | 100 | UD | UD |
| 85 | 78 | 0 | 400 | 100 | 0 | 100 | 0 | 0 | UD | UD |
| 86 | 79 | 0 | 0 | 0 | 0 | 0 | 100 | 0 | UD | UD |
| 87 | 80 | 400 | 200 | 800 | 0 | 100 | 100 | 100 | UD | UD |
| 88 | 83 | 400 | 200 | 800 | 0 | 100 | 100 | 0 | UD | UD |
